# Successful Treatment of Caesarean Scar Pregnancies by Local Treatment Only

**DOI:** 10.1155/2017/9543570

**Published:** 2017-03-15

**Authors:** Shinji Tanigaki, Chie Nagata, Kazunori Ueno, Nobuaki Ozawa, Shinichi Nagaoka, Kei Tanaka, Haruhiko Sago, Mitsutoshi Iwashita

**Affiliations:** ^1^Center for Maternal-Fetal, Neonatal and Reproductive Medicine, National Center for Child Health and Development, Tokyo, Japan; ^2^Department of Education for Clinical Research, National Center for Child Health and Development, Tokyo, Japan; ^3^Department of Obstetrics and Gynecology, Kawasaki Municipal Hospital, Kawasaki, Japan; ^4^Department of Obstetrics and Gynecology, Kyorin University School of Medicine, Tokyo, Japan

## Abstract

*Background*. Caesarean scar pregnancy (CSP) is a rare ectopic pregnancy associated with life-threatening complications. To date, no therapeutic protocols have been established. Sono-guided local methotrexate (MTX) injection is a relatively easy and low-invasive treatment. Additional systemic MTX is sometimes needed for CSP cases, especially when *β*-subunit human chorionic gonadotropin (*β*-hCG) levels are >20,000 mIU/ml at diagnosis. We report on six cases of CSP treated with local MTX injection, five of which received combined local treatment.* Methods*. Under intravenous anesthesia, six CSPs including a case with *β*-hCG levels >20,000 mIU/ml received MTX injection to the gestational sac. Five cases received gestational sac aspiration. Three cases had additional local potassium chloride injection and one case had a saline injection aiming at the fetal heart beat concurrent with MTX injection. MTX was administered weekly if *β*-hCG levels stayed beyond the expected values.* Outcomes*. All cases achieved *β*-hCG normalization without additional systemic MTX, with one case having a successful pregnancy after treatment.* Conclusion*. Sono-guided local MTX injection with concurrent local treatment might be a potentially effective approach for CSP cases. The accumulation of further cases is necessary to confirm this.

## 1. Introduction

Caesarean scar pregnancy (CSP) is an ectopic pregnancy implanted within the uterine scar of a previous caesarean section. The incidence of CSP has been estimated to be up to 1 in 1,800 pregnancies [1]. CSP is associated with life-threatening complications, such as massive hemorrhage and uterine rupture [2].

Various surgical and conservative treatments for CSP have been reported. Birch Petersen et al. reviewed 2037 CSP cases, noted complications in each treatment, and recommended five approaches: resection through a transvaginal approach, laparoscopy, uterine artery embolization (UAE) in combination with dilatation and curettage (D&C) and hysteroscopy, UAE in combination with D&C [3], and hysteroscopy. However, transvaginal sono-guided local methotrexate (MTX) injection was not recommended because of observed major complications and the need for additional systemic treatments [4]. In a past report, local MTX injection—systemic MTX—was administered especially for cases with *β*-subunit human chorionic gonadotropin (*β*-hCG) levels >20,000 mIU/ml at diagnosis [5]. Furthermore, if not surgically repaired, scar dehiscence may affect future pregnancies [6].

Despite some disadvantages, local MTX injection is less invasive and easier to administer compared to surgical treatments [7]. Furthermore, better treatment outcomes may be possible by combining local MTX injection with other techniques. We present our experience of six unruptured CSP cases, including one with *β*-hCG levels higher than 20,000 mIU/ml. We examined their clinical courses, additional local treatment, and pregnancy after treatment.

## 2. Case Presentation

### 2.1. Cases

Six cases of CSP are presented in Table 1. Mean maternal age was 37.7 ± 5.3 years. Four patients had undergone only one prior caesarean section, whereas two patients had three previous caesarean sections. Gestational age at diagnosis ranged from 6^+0^ to 7^+6^ weeks, and the levels of *β*-hCG at diagnosis ranged from 1,196 to 67,419 mIU/ml. Four cases showed a fetal heartbeat at diagnosis. None of the patients complained of severe abdominal pain or massive vaginal bleeding during the observation period.

All patients were treated according to the below schedule. Case 1 required a saline injection into the location of the fetal heart beat in addition to local MTX injection to stop the fetal heartbeat. In three cases (cases  2–4), local KCl injection (cases 2 and 3, 1 mEq; case 4, 1.5 mEq) was performed before local MTX injection. Given the enlarged size of the gestational sac, five of six cases (cases  1–5) received transvaginal ultrasound-guided gestational sac aspiration prior to local MTX injection, using the other lumen of the needle until the gestational sac disappeared on ultrasound.

**Figure 1:**
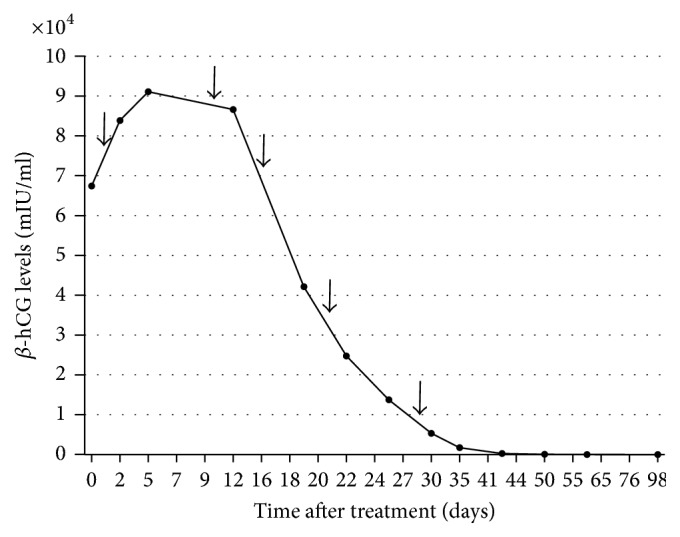
Curve of serum *β*-hCG levels for case 1. Arrow indicates MTX administration.

**Figure 2:**
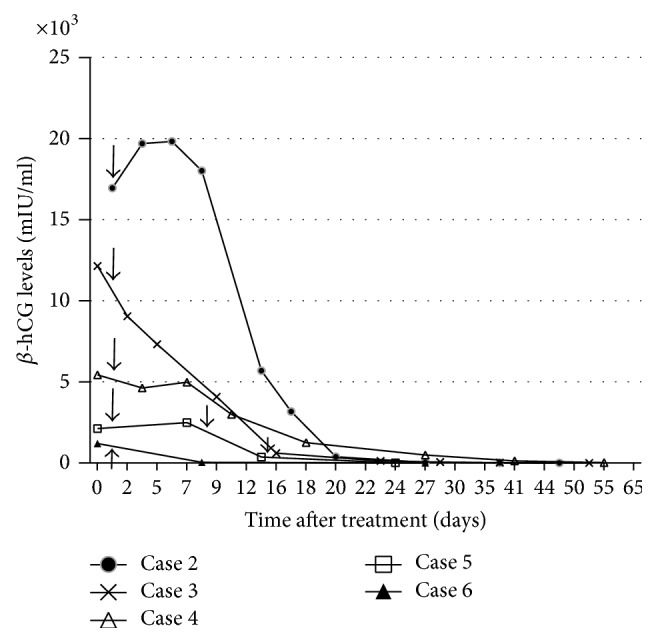
Curve of serum *β*-hCG levels for cases  2–6. Arrow indicates MTX administration.

Four patients were successfully treated with only one local treatment. Two patients required weekly MTX injection of 50 mg (case 1: 5 times; case 5: 3 times) because of elevated *β*-hCG levels after the first local treatment (Figures 1 and 2). The median duration from diagnosis of CSP to the time when *β*-hCG levels decreased to less than 5 mIU/ml was 52 ± 30 days. Considering the severity of case 1, we also calculated the duration excluding this case, which resulted in 43 ± 21 days. None of the patients required systemic MTX administration, operative procedures, uterine artery embolization, and/or blood transfusion. Estimated blood loss during the procedure was negligible. No postprocedure infection was found in any patient, and no uterine rupture occurred during the follow-up period. All patients stayed in the hospital for one day for the procedure, and prolonged hospitalization was not required for cases  2–6. Although case 1 had been admitted to hospital until her *β*-hCG levels started to decrease, only one-day admissions were needed for subsequent procedures.

After treatment for CSP, case 2 became pregnant spontaneously and had a good clinical course without complications. At 37^+0^ weeks' gestation, she went into labor and caesarean section was performed. She delivered a healthy 2,623 g female baby (Apgar score 7/8, 1 min/5 min). Operative findings showed that the bladder was densely adhered to the upper anterior uterus, and we could not incise the uterine lower segment. Although it was not visible due to adhesion with the bladder, dehiscence of the previous caesarean scar was palpable during surgery. Ultrasound examination on the 7th postpartum day did not reveal dehiscence, although repair was not conducted during surgery.

### 2.2. Diagnosis

**Figure 3:**
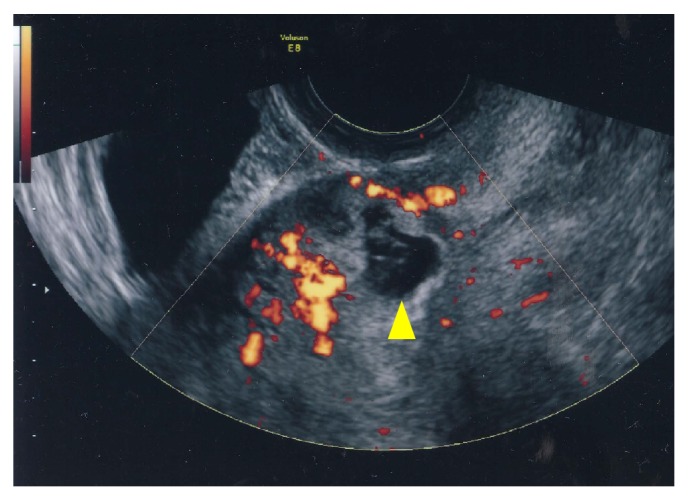
Transvaginal sonographic imaging. A midline sagittal view presenting the gestational sac at the uterine scar of a previous caesarean section and empty uterine cavity (power Doppler study). Triangle indicates the gestational sac.

Six cases of CSP were diagnosed at two of our affiliated institutions between 2008 and 2013. The diagnoses were made by two authors, ST (cases 1, 5, and 6) and KU (cases  2–4), using ultrasound (Volson E8, GE Healthcare, Japan). All cases fulfilled the criteria for sonographic diagnosis of CSP developed by Godin et al., which are (1) an empty uterine cavity and no contact with the gestational sac; (2) an empty cervical canal that is clearly visible and has no gestational sac or ballooning at early diagnosis; (3) presence of the gestational sac with or without a fetal pole and with or without fetal cardiac activity in the anterior part of the uterine isthmus; and (4) no myometrial tissue or a defect in the myometrial tissue between the bladder and gestational sac [8] (Figure 3).

### 2.3. Treatment Schedule

Written informed consent was obtained from all six patients before transvaginal sono-guided local MTX injection. After overnight fasting and emptying of the bladder, the patients underwent the procedure in the lithotomy position under intravenous anesthesia. After disinfecting the vagina with 5% iodine, the myometrium around the gestational sac was punctured with a 16-gauge double-lumen needle under the guidance of transvaginal ultrasound (Sonovista 2000, Mochida Siemens Medical, Japan). Depending on the size of the gestational sac, transvaginal ultrasound-guided gestational sac aspiration was performed using the other lumen of the needle until the gestational sac disappeared on ultrasound. MTX (50 mg/body) dissolved in 2 ml of distilled water was injected through the lumen of the needle followed by saline injection. Addition local injection of KCl (1 mEq~1.5 mEq) was administered whenever necessary. Local MTX injection of 50 mg was administered weekly if the hCG levels stayed beyond the expected values.

### 2.4. Follow-Up Schedule

Patients' serum *β*-hCG levels were examined every 2 to 5 days and ultrasound scans were undertaken until patients' *β*-hCG levels became less than 5 mIU/ml. Serum *β*-hCG levels independent of serum LH levels were measured by CLEIA (cases  1, 5, and 6) or FEIA (cases  2–4) methods.

## 3. Discussion

All cases were able to achieve *β*-hCG normalization without additional systemic MTX administration or surgical treatments. This is the first report to show that all CSP cases, including one with *β*-hCG levels higher than 20,000 mIU/ml, were successfully treated using local treatment without additional systemic MTX. To ensure successful preservation of fertility and to avoid uterine rupture, additional systemic MTX was administrated in previous cases [7]. In particular, when the *β*-hCG level at the time of diagnosis was higher than 20,000 mIU/ml, or when *β*-hCG level after local MTX injection was elevated, additional systemic MTX administration was performed [5]. However, systemic MTX administration can cause complications, such as nausea, stomatitis, alopecia, bone marrow, depression, and pneumonitis [9]. Therefore, it would be beneficial if local treatment without systemic MTX was sufficient to treat CSP with higher *β*-hCG levels.

It is noteworthy that local MTX injection may be difficult to administer when the gestational sac is large and *β*-hCG levels are higher than 20,000 mIU/ml. Transvaginal sono-guided gestational sac aspiration allows for easier administration of local MTX injection by decreasing the volume of the gestational sac. Concurrent saline injection to the location of the fetal heart beat and/or KCl into the fetal body also increase the curative effect by decreasing fetal viability. Additionally, these injections may reduce the number of times that local MTX injection is required. Notably, using a double-lumen needle allows for easier performance and avoids the need for additional puncture. To decrease fetal viability, bilateral uterine artery chemoembolization with MTX was reported to be effective. However, facilities that provide this type of treatment are limited. Furthermore, the impact of bilateral uterine artery chemoembolization with MTX on future pregnancy is unclear and the length of hospital stay was reportedly longer (4–28 days) [10].

In this study, one case eventually achieved successful delivery after treatment. However, we observed uterine scar dehiscence on palpation at delivery. There are only a few reports on pregnancy after conservative treatment for CSP. Maymon et al. reported two cases of pregnancy after treatment of CSP with only conservative therapy. One patient miscarried at 17 weeks [11]. Since the impact of unrepaired scar dehiscence on future pregnancies and the effect of MTX on the scar are unclear, further study in this area is required. In conclusion, we have shown the possibility of treating CSP with sono-guided local MTX injection and concurrent local treatment, such as gestational sac aspiration, saline injection into the location of the fetal heartbeat, and KCl injection into the gestational sac, even when *β*-hCG levels are higher than 20,000 mIU/ml at diagnosis. Nevertheless, the accumulation of further cases is necessary to validate this treatment modality.

## Figures and Tables

**Table 1:** Clinical data of patients with caesarean scar pregnancy.

Case	Age (years)	Number of previous caesarean sections	Gestational age at diagnosis (weeks)	Gestational age at start of treatment (weeks)	*β*-hCG level at diagnosis (mIU/ml)	Maximum *β*-hCG level (mIU/ml)	Fetal heart beat (yes/no)	Number of times of local MTX administration	Gestational sac aspiration (yes/no)	Concurrent local injection	Time after treatment for *β*-hCG levels to decrease to <5 mIU/ml (days)	Time after treatment for menstruation to return (days)	Pregnancy after treatment (yes/no)
1	39	3	6 + 6	7 + 1	67419	91087	Yes	5	Yes	Saline injection into the location of the fetal heart beat	98	180	No
2	35	1	7 + 2	7 + 4	16946	19693	Yes	1	Yes	KCl of 1 Meq into gestational sac	36	48	Yes
3	41	3	7 + 4	7 + 6	12137	12137	Yes	1	Yes	KCl of 1 Meq into gestational sac	51	43	No
4	28	1	6 + 0	6 + 1	5427	5427	Yes	1	Yes	KCl of 1.5 Meq into gestational sac	76	70	No
5	41	1	6 + 4	7 + 0	2119	2485	No	3	Yes	None	24	Unclear	No
6	42	1	7 + 6	8 + 1	1196	1196	No	1	No	None	27	64	No
